# Metabolomics Applications for Diagnosing Peri-Implantitis: A Systematic Review of In Vivo Studies

**DOI:** 10.3390/diagnostics15080990

**Published:** 2025-04-14

**Authors:** Ana-Maria Condor, Andreea Kui, Daniela Cornelia Condor, Marius Negucioiu, Smaranda Dana Buduru, Patricia Ondine Lucaciu

**Affiliations:** 1 Oral Health Discipline, Department 3—Oral Rehabilitation, Faculty of Dental Medicine, “Iuliu Hațieganu” University of Medicine and Pharmacy, 400012 Cluj-Napoca, Romania; ana.mari.condor@elearn.umfcluj.ro (A.-M.C.);; 2 Cluj County Emergency Clinical Hospital, 400006 Cluj-Napoca, Romania; 3 Prosthodontics Discipline, Department 4—Prosthodontics and Dental Materials, Faculty of Dental Medicine, “Iuliu Hațieganu” University of Medicine and Pharmacy, 400012 Cluj-Napoca, Romania; 4 Periodontology Discipline, Department 3—Oral Rehabilitation, Faculty of Dental Medicine, “Iuliu Hațieganu” University of Medicine and Pharmacy, 400012 Cluj-Napoca, Romania

**Keywords:** peri-implantitis, peri-implant crevicular fluid, metabolomics, diagnosis

## Abstract

**Background/Objectives**: Peri-implantitis is a prevalent inflammatory condition affecting dental implants, leading to increased treatment costs, patient dissatisfaction, and potential implant failure. Novel biomarker-based approaches may contribute to early detection, thereby decreasing the burden of the disease. The aim of this review was to assess in vivo studies using metabolomics to identify the metabolic profiles and potential biomarkers of peri-implantitis. **Methods**: The protocol for this study was registered with PROSPERO (CRD42025634865). Five databases and grey literature sources (PubMed, Scopus, Web of Science, ProQuest, and Google Scholar) were searched using keywords related to metabolomics and peri-implantitis. Studies were selected by independent, inter-calibrated researchers. Data were extracted using predefined, custom forms. The risk of bias was assessed using the ROBINS-I tool. **Results**: An electronic literature search retrieved 543 articles, of which five were selected. All studies were published within the last five years of the search. All but one study used untargeted metabolomics, and all studies identified metabolites associated with peri-implantitis or distinct metabolomic profiles of peri-implantitis. SCFAs and lysine metabolites were recurring in the results, confirming the findings of previous metabolomic studies on periodontal disease. **Conclusions**: Metabolomics has not been widely used to study peri-implantitis. Evidence from existing studies confirms the findings of metabolomics studies on periodontitis. Several metabolites related to PI are associated with immune response, tissue degradation, and cellular energy pathways. Integrating -omics technologies into peri-implantitis diagnosis may facilitate biomarker discovery and improve early detection strategies.

## 1. Introduction

Dental implant therapy is one of the most frequent approaches in dentistry and is used to treat partial or complete edentation cases. As life expectancy continues to grow, so do the challenges of maintaining the health and integrity of oral structures; hence, the number of cases treated with implants is increasing [1,2]. Peri-implantitis is a significant clinical challenge in dental implantology. Dental implant therapy is widely used to address edentulism and maintain oral function; however, the increasing prevalence of complications such as peri-implantitis compromises implant longevity and oral tissue integrity [1,2]. However, managing complications such as peri-implantitis remains a significant challenge [3,4]. Implant complications are common and may impose high financial burdens on patients, leading to dissatisfaction and negative treatment perceptions [5,6]. This is particularly relevant when considering patients’ elevated expectations of implant therapy as a permanent solution for oral or dental issues [7].

Implant complications and deficiencies in the surrounding tissues may be a consequence of mechanical, biological, systemic, traumatic, or iatrogenic factors [8]. Peri-implant diseases (mucositis—PIM and peri-implantitis—PI) are the most relevant biological complications, with PI occurring in 19.5% of patients and 12.5% of implants [9]. A systematic review estimated the prevalence of peri-implantitis at 43% and peri-implant mucositis at 22% across Europe, North America, and South America, with an increasing incidence over time [10]. If left untreated, peri-implantitis may lead to implant loss as early as 2 months after the initial diagnosis [4,11,12,13]. This inflammatory condition is characterised by accelerated bone loss and soft tissue destruction, often leading to implant failure if left untreated [4,11,12,13]. The biological and mechanical factors contributing to peri-implantitis are multifactorial, including microbial dysbiosis, patient-related risk factors, and the complex interplay of host immune responses, which collectively increase the risk of achieving long-term treatment success [3,8]. However, PI treatment is associated with considerable challenges. A primary concern is patient compliance with oral hygiene practices and implant maintenance. Furthermore, surgical treatment of PI may lead to major aesthetic deficiencies due to subsequent soft tissue retraction [14]. Most importantly, PI treatment is only moderately successful in the short term. A large number of cases (75%) recur or remain unresolved after five years [15]. A recent systematic review reported that fewer than 50% of implants affected by PI achieve disease resolution [16]. PI is triggered and maintained by numerous factors, some of which are difficult to manage. Therefore, due to the unpredictability of treatment success, rigorous prevention measures should be taken to avoid PI whenever possible [17].

Beyond its direct clinical implications, peri-implantitis significantly affects the overall well-being of patients. Affected individuals frequently experience chronic discomfort, reduced masticatory efficiency, and aesthetic deficiencies, which can ultimately diminish the oral health-related quality of life [5,6]. The potential for rapid progression to implant loss and the ensuing need for extensive, costly re-treatment not only intensifies physical discomfort but also contributes to psychological stress and increased financial burden [7,14]. By highlighting these patient-centered consequences, the urgency for early, non-invasive diagnostic methods, such as metabolomic profiling, is underscored, promising improved personalised management strategies that address both clinical and quality-of-life outcomes.

The current PI diagnostic criteria are based on clinical and radiological measurements compared to the baseline values. These include inflammation, bleeding, and/or suppuration upon probing, increased probing depth (PD), and bone loss (BL) [11]. Unfortunately, PI follows a non-linear and accelerating pattern and can occur early during post-surgical follow-up. Therefore, permanent damage to the supporting tissues is frequently observed at the time of diagnosis. This emphasises the need for novel, non-invasive early detection methods for peri-implantitis [14,18,19]. The application of emerging omics technologies in the diagnostic process is essential due to the significant challenges posed by PI therapy and its increasing burden on patients. Integrating -omics technologies into dental practice contributes to the development of precision oral healthcare and personalised treatment [19]. Metabolomics is a powerful tool since it best reflects the molecular phenotype of a sample at the time of analysis [20]. Various molecules (such as cytokines and enzymes) have been previously validated as markers for peri-implantitis [21,22,23], but no metabolic biomarkers have been identified to date.

Metabolomics could facilitate the early diagnosis, prognosis, and monitoring of PI by non-invasively sampling peri-implant crevicular/sulcular fluid (PICF/PISF). It can detect and quantify metabolites associated with bacterial dysbiosis or incipient disease states, thus preventing large-scale irreversible tissue damage. Integrating metabolic data with other omics or microbiological approaches could contribute to a better understanding of this pathological entity. This could provide opportunities for new preventive strategies and treatment options [24,25,26]. The objective of this review was twofold: firstly, to assess existing in vivo studies on metabolomics applied in the diagnosis, prognosis, or treatment of peri-implantitis, and secondly, to identify metabolites mentioned in two or more studies, which could represent the subject of future research.

## 2. Materials and Methods

### 2.1. Registration and Validation of Study Protocol

This review was developed using the Preferred Reporting Items for Systematic Reviews and Meta-Analyses (PRISMA) 2020 reporting guidelines and PRISMA for abstracts [27]. The study protocol was registered with the International Prospective Register of Systematic Reviews (PROSPERO CRD42025634865).

### 2.2. Question of Study

How can metabolomics technology (metabolic profiling) be applied to the diagnosis, prognosis, or treatment of peri-implantitis in human patients suffering from peri-implantitis?

### 2.3. Eligibility Criteria

a. Inclusion criteria
  P (population) = patients treated with dental implants;
  E (exposure) = clinically diagnosed peri-implantitis;
  C (control) = implants in a state of clinically determined peri-implant health
  O (type of outcome measures) = differences in detectable metabolites from saliva of peri-implant crevicular fluid samples, assessed by both targeted or untargeted metabolomics approaches, marginal bone loss, bleeding on probing, and probing depth.
  S (type of studies) = original studies on humans, RCT, NRCT, prospective, retrospective, or cross-sectional studies, case reports and case series, and cohort studies
b. Exclusion criteria
  - Study designs: literature reviews and/or meta-analyses, letters to editors, conference abstracts, and commentaries.
  - In vitro, animal study designs, and ex vivo studies.
  - Studies without full-text articles.
  - Studies that presented missing or incomplete data regarding outcome measures or the technologies and pathologies involved.
  - Studies published in languages other than English.

### 2.4. Search Strategy

The existing literature was electronically searched using the following databases and registries: PubMed, Scopus, Web of Science, ProQuest, and Google Scholar. The electronic literature search was designed and conducted by two independent researchers (AMC and CDC), starting from 21 January 2025 until the final date of 30 January 2024. The search strategy included the terms ‘peri-implantitis’ and ‘metabolomics’, applied using Boolean operators (AND/OR) in title, abstract, and full-text searches. When available, MeSH terms were included in the search. No date restrictions or language restrictions were applied during the search. This strategy was applied with minor necessary modifications to the queries to accommodate the search particularities and controlled vocabularies of each database. The exact search terminologies are available in Appendix A—Search strategy.

### 2.5. Study Selection Process

Search results were downloaded in library form from each database (when possible) and centralised using a reference manager (Zotero version 7.0.6.). Duplicates were identified manually and electronically using the same software. The remaining studies were screened for titles and abstracts by 2 researchers and selected based on the inclusion and exclusion criteria. Studies considered relevant were retrieved in full-text form and selected by 2 independent researchers. For databases that had no option of downloading results, the articles were manually selected based on title/abstract and then retrieved in full-text form by the same 2 researchers. Conflicts were resolved by a third researcher. Prior to the selection process, the researchers inter-calibrated and trained for selection on a batch of 100 random results. The inter-researcher’s agreement level was calculated by the Kappa coefficient (k = 9.1)

### 2.6. Data Extraction

The following data were extracted from the included studies using custom, predefined forms:

- General data about the studies (title, main authors, geographical area, DOI, year of publication, study design)
- Population (number of subjects/number of implants, age/gender distribution, personal potential confounding factors)
- Exposure and controls (marginal bone loss, probing depth, bleeding on probing, other periodontal or peri-implant indexes)
- Outcome (metabolomics approaches used, technologies involved, sample types, identified metabolites)

### 2.7. Risk of Bias/Quality Assessment

ROBINS-I (“Risk Of Bias In Non-randomised Studies—of Interventions”) scale was used to assess the risk of bias. This scale assesses the risk of bias in 7 domains: bias due to confounding, study participants’ selection, classification of interventions, deviations from intended interventions, missing data, measurements of outcomes, and reporting. Each domain was assessed using the tool guidelines and summarised under the labels proposed by the tool. To generate a visual summary of the assessment, we used the robvis tool to generate a traffic-lights graph [28,29].

## 3. Results

### 3.1. Study Selection

**Figure 1:**
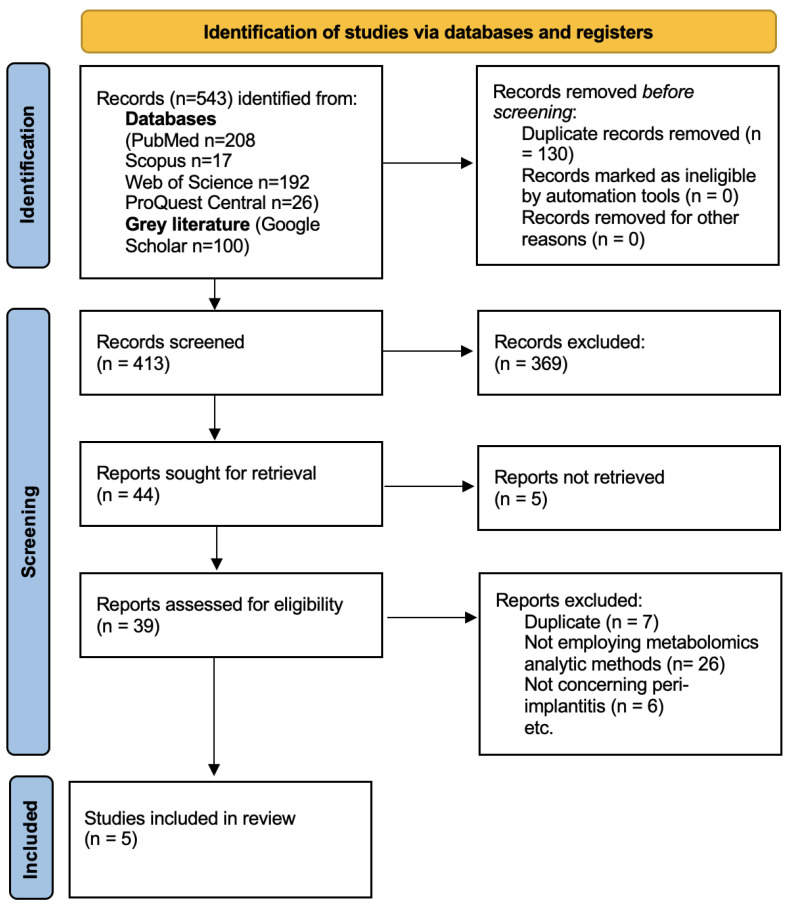
PRISMA 2020 flowchart of the study selection process.

The search retrieved 543 results. After duplicate removal, 413 articles remained. The screening and study selection processes are summarised in the following PRISMA 2020 flowchart (Figure 1). A total of five studies were included in this review.

### 3.2. Description of Included Studies

Two of the included studies were theses published in 2020 [30] and 2021 [31]. Two studies were published in 2024 [32,33] and one study was published in 2023 [34]. 2 studies employed H-NMR [30,31], one study SERS [34] and two studies LC and GC [32,33]. All studies analysed peri-implant crevicular (sulcular fluid) samples. All studies clinically diagnosed PI. Excepting for one study [32] based on targeted metabolomics, all studies used untargeted metabolomics. A detailed description of included studies is available in Table 1.

### 3.3. Identified Metabolites

The most commonly identified metabolites were consistently associated with peri-implant inflammation and tissue degradation. The following metabolites were reported in studies: isobutyric acid [32,33] and propionic acid [30,32] (short-chain fatty acids or SCFAs), valine [30,33], proline, and cadaverine/lysine [30,31] (amino acids), hypoxanthine [33,34] (a purine), and alpha-ketoglutarate [30,31] (an intermediate in the Krebs cycle).

PI was correlated with SCFAs (propionate, butyric acid, isobutyric acid, isovaleric acid, and succinic acid), carbohydrate derivatives (fructose-6-phosphate and glucose-6-phosphate), amino acids (lysine, alanine, threonine, and valine), polyamines (cadaverine and putrescine), antioxidants (glutathione and ergothioneine), purines (hypoxanthine), and monoamines (tyramine). SCFAs were also correlated with increased periodontal parameters (isobutyric and propionic acids) and PIM (formic, acetic, propionic, and isovaleric acids). Amino acids were also correlated with increased probing depth (phenylalanine, valine), accelerated bone loss (proline), and stabilised PI (arginine).

### 3.4. Risk of Bias/Quality Assessment of the Studies

**Figure 2:**
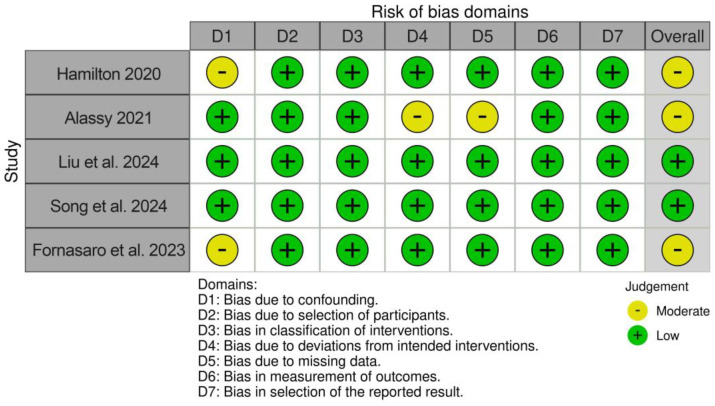
Results of risk of bias/quality assessment [30,31,32,33,34].

The risk of bias assessment revealed two studies with a low risk of bias [32,33] and three studies with a moderate risk of bias [30,31,34]. The results are displayed in Figure 2.

## 4. Discussion

### 4.1. Main Findings and Interpretation in the Context of Available Literature

Omics-based technologies provide insights into the molecular mechanisms and shifts between healthy and diseased states. Despite limited research, preliminary findings suggest that metabolomics can distinguish the metabolic profiles of healthy and diseased implants, offering the potential for early diagnosis. Current applications of metabolomics in dental research are focused on oral cancer [35,36,37], periodontitis [38,39,40], and caries detection [41,42,43], with little available research focused on PI or PIM. The most common sample types include saliva, gingival crevicular fluid (GCF), microbial plaque, and oral tissues. Metabolites are low-molecular-weight molecules which represent the end-products of metabolic processes and, in contrast to other -omics approaches, directly reflect the biochemical activity and state of tissues in their profile and concentrations. They can be endogenous (produced by the host organism and its microbiota) or exogenous (from diet, environment, and lifestyle) [44,45,46]. Metabolomics analysis can be conducted in two main directions: targeted and untargeted. Targeted metabolomics measures a defined set of previously characterised metabolites, while untargeted metabolomics comprehensively analyses all detectable metabolites in a sample, including unknown metabolites [47]. Lipidomics is a branch of targeted metabolomics that studies the interactions of lipids in disease processes. Two main analytical platforms are used in metabolomics: mass spectrometry (MS), frequently combined with liquid or gas chromatography (LC or GC), and nuclear magnetic resonance (NMR) spectroscopy [45]. Surface-enhanced Raman spectroscopy (SERS) is a rapid and accurate emerging method for metabolite detection [48,49].

Peri-implant diseases present shared characteristics with periodontal diseases. Both involve dysbiotic biofilms, which trigger host defence immune pathways and lead to chronic inflammation [50]. Studies have reported differences in the bacteriomes of PI and periodontitis, although a distinct microbial profile of peri-implantitis has not yet been established [51]. However, periodontitis is a risk factor for peri-implantitis development. Bacterial colonisation from periodontitis sites to peri-implant sites is frequent [51,52,53,54]. Consequently, comparing the results of periodontitis and peri-implantitis metabolomics studies may help validate the initial findings.

No metabolites recurred in more than two studies, probably due to the small number of included studies. The studies by Hamilton [30] and Alassy [31] had three metabolites in common, likely because both studies used H-NMR and similar protocols. The recurrent metabolites identified in all the included studies are discussed further in this section.

Isobutyric acid and propionic acid belong to the group of short-chain fatty acids (SCFAs). SCFAs are produced by gut and oral bacteria. In the oral cavity, they are produced primarily in periodontal pockets by periodontal pathogens like *P. gingivalis* and *Fusobacterium Nucleatum* [55]. Isobutyric acid is an isomer of butyrate. Butyrate has been frequently associated with periodontitis in various metabolomics studies [39,56,57,58]. It maintains bacterial metabolism and promotes bacterial growth by increasing haeme production. Furthermore, it has destructive effects on the periodontal tissues. In human gingival epithelial cells (HGECs), it promotes cell apoptosis and has a destructive effect on intercellular junctions. In human gingival fibroblasts (HGFs), it promotes cell death and the release of pro-inflammatory cytokines. These cytokines include interleukin-1 beta, interleukin-6, and tumour necrosis factor alpha, which are established biomarkers for periodontal diseases. Therefore, butyrate contributes to promoting and maintaining chronic inflammation [55,59,60]. This validates the findings of Song et al. [33] and Liu et al. [32].

Cadaverine is a metabolite of lysine. Lysine is an essential amino acid whose levels are depleted by pathogenic bacteria during periodontal destruction. Cadaverine has been previously associated with increased periodontal inflamed surface areas (PISA) and periodontitis [61,62,63,64,65,66]. The association of cadaverine with PI could be linked to both bacterial metabolism and tissue degradation. Both Alassy [31] and Hamilton [30] identified cadaverine in association with PI.

Valine is an essential proteinogenic amino acid involved in stress, energy, and muscle metabolism. Previous studies have associated it with periodontitis, with fluctuating levels [56]. Under experimental conditions, certain concentrations of valine inhibited *P. gingivalis* biofilm formation and affected bacterial polysaccharide production [67]. While Hamilton [30] identified valine as a marker for PI, Song et al. [33] correlated it with increased probing depths.

Hypoxanthine is a purine derivative. It is a chemical intermediate in the production of nucleic acids and the metabolism of adenosine. Purine degradation and reactive oxygen species production are accelerated in disease contexts, suggesting that higher levels of hypoxanthine may be linked to periodontal destruction [68,69,70]. Hypoxanthine has been associated with PI in the study by Fornasaro et al. [34] and was significantly correlated with bleeding on probing in the study by Song et al. [33].

Proline is a proteogenic amino acid and a component of collagen. Collagen can be found in the extracellular matrix, connective tissues, and bone. Proline molecules can be rapidly released during inflammation by the sequential action of matrix metalloproteinases, peptidases, and prolidase [71]. Extracellular proline increases the rate of collagen production, suggesting a possible connection between proline and healthy implants [72,73,74]. However, proline is also a marker of tissue degradation. It can be used as a precursor for superoxide radicals, which initiate the process of cellular and extracellular apoptosis [69]. This could potentially explain the correlation between increased levels of proline and periodontitis [40,61,75].

Alpha-ketoglutarate is an intermediate metabolite of the Krebs cycle, which regulates ATP production and contributes to oxidative stress defence. It is indispensable for amino acid and protein synthesis [76,77]. Furthermore, it has a proven anabolic effect on bone metabolism and is associated with bone homeostasis, improving osseointegration of dental implants [77,78,79]. This confirms the findings of Alassy and Hamilton [30,31], who concluded that alpha-ketoglutarate is associated with healthy implants.

### 4.2. Study Limitations and Strengths

This review was conducted according to a pre-registered protocol submitted to PROSPERO. It adhered to the PRISMA-2020 guidelines for systematic reviews, ensuring accuracy, transparency, and methodological rigour. To the best of our knowledge, this is the first review of metabolomics applied to the diagnosis of peri-implant disease. An electronic literature search was conducted in numerous databases, including grey literature. This ensured an elaborate and comprehensive approach to identify relevant studies and provide a broad overview. Metabolomics is a novel approach in peri-implantitis research, and there is a scarcity of studies on this subject. Because of the small number of included studies and the variety of approaches used, a meta-analysis could not be conducted. However, the risk of bias assessment results suggest a high degree of study quality. In addition, the findings of the included studies confirmed the results of previous studies on periodontitis. This review provides an initial evaluation of the literature and a starting point for developing new research on metabolomic approaches in peri-implantitis, contributing to periodontics and dentistry.

### 4.3. Clinical Implications

Based on our research, the integration of metabolomics into clinical practice has significant potential to improve the management of peri-implantitis. By facilitating non-invasive early detection through specific metabolic biomarkers, clinicians can identify peri-implant inflammation at subclinical stages, allowing for timely intervention and potentially preventing irreversible tissue damage. This early diagnostic capability is in line with the principles of personalised dentistry, allowing tailored preventive strategies based on an individual’s unique metabolic profile, thus reducing both treatment complexity and associated costs.

In addition, metabolomics could serve as a practical tool for the prognosis and monitoring of therapeutic outcomes, guiding personalised treatment decisions. Regular monitoring of peri-implant crevicular fluid metabolites could provide clinicians with objective, real-time insights into the progression or resolution of peri-implant disease, thereby enabling more precise and effective therapeutic interventions. Eventually, incorporating metabolomic analysis into peri-implant care could lead to improved patient outcomes, reduce treatment burden, and increase patient satisfaction through personalised, targeted preventive strategies.

## 5. Conclusions

Although current evidence is limited, metabolomics shows promise for the diagnosis of peri-implantitis. This technology is increasingly used to study periodontitis, helping to understand its development, biological mechanisms, and progression. In particular, small molecules (metabolites), such as short-chain fatty acids and amino acids, have been frequently identified in relation to disease characteristics, such as periodontal inflammation and tissue degradation. Consequently, metabolomics could provide insights into the differences and similarities between periodontal and peri-implant diseases. Combining metabolomics with other approaches from the -omics spectrum (such as genomics, transcriptomics, and proteomics) is desirable and could provide a deeper understanding of these pathologies. Future research should focus on standardising study protocols, integrating multi-omics platform data, and validating identified metabolites and key findings in larger clinical trials.

## Figures and Tables

**Table 1:** Description of the included studies.

Main Author	Year	Analytic Platform	Approach	Study Type	Sample Type	Sample Size	Inclusion Criteria	Metabolites Identified (nr)	Significant Metabolites	Notes	Conclusions
Song et al. [33]	2024	UHPLC-MS, GC-MS	Targeted and untargeted	Cross-sectional	PICF	56 patients (56 implants)	>18 years old; minimum 12 months functional loading, clinical diagnosis of PI/IH	179	succinic acid, fructose-6-phosphate, glucose-6-phosphate (correlated to PI and periodontal parameters); Isobutyric acid, 3-phenylpropionic acid (correlated with periodontal parameters); L-phenylalanine, benzamide, and L-valine (correlated to PD); Photoline, chlorphenguanidine, pyrrolidine, and hypoxanthine (correlated to BOP)	Some participants in the peri-implantitis group also had periodontitis.	A distinct metabolite profile between healthy implants and implants affected by peri-implantitis exists, and it correlates with existing bacterial flora.
Alassy [31]	2021	H-NMR	Untargeted	Longitudinal	PICF; saliva	71 patients (130 implants)	Good general health, clinical diagnosis of PI/IH	36	Cadaverine/lysine (correlated to PI); alpha-ketoglutarate (correlated to healthy implants); Proline and 1-3-diamino propane (predictors for bone loss >1 mm); arginine (associated with non-progressive PI)	Study included smoking patients;	PI PICF samples demonstrate a distinct metabolic profile compared to healthy implants.
Fornasaro et al. [34]	2023	SERS	Untargeted	Cross-sectional	PICF	118 patients (305 implants)	>18 years old; minimum 12 months functional loading, good general health, clinical diagnosis of PI/HI	NR	glutathione, ergothioneine, and hypoxanthine	Study included patients diagnosed with peri-mucositis	SERS could be a non-invasive tool to monitor implant health and PI development
Hamilton [30]	2020	H-NMR	Untargeted	Longitudinal	PICF	59 patients (128 implants)	Good general health with controlled systemic diseases, clinical diagnosis of PI/HI	35	Cadaverine/lysine, propionate, alanine/lysine, putrescine/lysine, valine, tyramine, and threonine (correlated to PI); a- ketoglutarate, isoleucine, proline, and uracil (correlated to implant health)	Study included patients diagnosed with peri-mucositis	Specific metabolites were significantly correlated with PI or HI but showed insufficient sensitivity/specificity to diagnose PI.
Liu et al. [32]	2024	GC-MS, HPLC	Targeted	Cross-sectional	PICF	48 patients (86 implants)	>18 years of age; minimum 5 years since implant placemen; no mechanical complications of implants; clinically diagnosed PI, PIM or PH	NA	Formic acid, acetic, propionic, and isovaleric acids (correlated to PIM); butyric, isobutyric, and isovaleric acids (correlated to PI)	Study searched specifically for SCFAs	Short-chain fatty acids were significantly correlated with PI clinical parameters. Elevated specific SCFAs are correlated with peri-implant disease.

Abbreviations (in order of appearance): UHPLC-MS = ultra high-performance liquid chromatography coupled with mass spectrometry; GC-MS = gas chromatography coupled with mass spectrometry; PICF = peri-implant crevicular fluid; H-NMR = proton nuclear magnetic resonance; PI = peri-implantitis; HI = healthy implants; SERS = surface-enhanced Raman spectroscopy; HPLC = high-performance liquid chromatography; PIM = peri-implant mucositis; SCFAs = short-chain fatty acids.

## Appendix A. Search Strategy

**Database/Registry**	**Query**	**No. of Results**	**Date**
PubMed	(“peri implantitis”[MeSH Terms] OR “peri implantitis”[All Fields] OR “periimplantitis”[All Fields] OR (“dental implants”[MeSH Terms] OR (“dental”[All Fields] AND “implants”[All Fields]) OR “dental implants”[All Fields] OR (“dental”[All Fields] AND “implant”[All Fields]) OR “dental implant”[All Fields]) OR (“periimplant”[All Fields] AND (“disease”[MeSH Terms] OR “disease”[All Fields] OR “diseases”[All Fields] OR “disease s”[All Fields] OR “diseased”[All Fields])) OR (“peri-implant”[All Fields] AND “crevicular”[All Fields] AND (“fluid”[All Fields] OR “fluid s”[All Fields] OR “fluids”[All Fields])) OR “PICF”[All Fields]) AND (“high-performance liquid chromatography”[Text Word] OR “mass spectrometry”[Text Word] OR “nuclear magnetic resonance spectroscopy”[Text Word] OR “liquid chromatography”[All Fields] OR “gas chromatography”[All Fields] OR (“metabolome”[MeSH Terms] OR “metabolomics”[MeSH Terms]) OR “metabolom*”[All Fields] OR “lipidomic*”[All Fields])	208	21 January 2025
Scopus	TITLE-ABS-KEY (peri-implantitis OR periimplantitis OR “dental implant*” OR peri-implant AND disease OR peri-implant AND crevicular AND fluid OR “PICF”) AND TITLE-ABS-KEY (metabolom* OR metabolomics OR metabolome OR lipidom* OR metabolite* OR “high-performance liquid chromatography” OR “mass spectrometry” OR “nuclear magnetic resonance spectroscopy” OR “liquid chromatography” OR “gas chromatography”)	17	21 January 2025
Web of Science	ALL=(peri-implantitis OR periimplantitis OR “dental implant*” OR peri-implant AND disease OR peri-implant AND crevicular AND fluid OR “PICF”) AND ALL=(metabolom* OR metabolomics OR metabolome OR lipidom* OR metabolite* OR “high-performance liquid chromatography” OR “mass spectrometry” OR “nuclear magnetic resonance spectroscopy” OR “liquid chromatography” OR “gas chromatography”)	192	21 January 2025
ProQuest Central	summary((peri-implantitis OR periimplantitis OR (“dental implant” OR “dental implantology” OR “dental implants”) OR peri-implant AND disease OR peri-implant AND crevicular AND fluid OR “PICF”) AND (metabolom* OR metabolomics OR metabolome OR lipidom* OR metabolite*))	26	21 January 2025
Google Scholar	peri-implantitis periimplantitis “dental implant*” peri-implant disease peri-implant crevicular fluid PICF metabolom* metabolomics metabolome lipidom* metabolite* high-performance liquid chromatography “mass spectrometry” “nuclear magnetic resonance spectroscopy” 1H NMR “liquid chromatography” “gas chromatography”Results were sorted “by relevance” and the first 10 pages of results were considered	100	21 January 2025
Total		543	

## References

[1] Misch C.E. (2014). Rationale for dental implants. Dental Implant Prosthetics.

[2] Srinivasan M., Meyer S., Mombelli A., Müller F. (2017). Dental implants in the elderly population: A systematic review and meta-analysis. Clin. Oral Implant. Res..

[3] Mauer R.G., Shadrav A., Dashti M. (2021). Predictability of Dental Implants. Innovative Perspectives in Oral and Maxillofacial Surgery.

[4] Berglundh T., Jepsen S., Stadlinger B., Terheyden H. (2019). Peri-implantitis and its prevention. Clin. Oral Implants Res..

[5] Wang Y., Bäumer D., Ozga A.K., Körner G., Bäumer A. (2021). Patient satisfaction and oral health-related quality of life 10 years after implant placement. BMC Oral Health.

[6] Pradyachaipimol N., Tangsathian T., Supanimitkul K., Sophon N., Suwanwichit T., Manopattanasoontorn S., Arunyanak S.P., Kungsadalpipob K. (2023). Patient satisfaction following dental implant treatment: A survey. Clin. Implant. Dent. Relat. Res..

[7] Abrahamsson K.H., Wennström J.L., Berglundh T., Abrahamsson I. (2017). Altered expectations on dental implant therapy; views of patients referred for treatment of peri-implantitis. Clin. Oral Implants Res..

[8] Hämmerle C.H.F., Tarnow D. (2018). The etiology of hard- and soft-tissue deficiencies at dental implants: A narrative review. J. Clin. Periodontol..

[9] Diaz P., Gonzalo E., Villagra L.J.G., Miegimolle B., Suarez M.J. (2022). What is the prevalence of peri-implantitis? A systematic review and meta-analysis. BMC Oral Health.

[10] Derks J., Tomasi C. (2015). Peri-implant health and disease. A systematic review of current epidemiology. J. Clin. Periodontol..

[11] Renvert S., Persson G.R., Pirih F.Q., Camargo P.M. (2018). Peri-implant health, peri-implant mucositis, and peri-implantitis: Case definitions and diagnostic considerations. J. Clin. Periodontol..

[12] Polymeri A., Loos B.G., Aronovich S., Steigmann L., Inglehart M.R. (2022). Risk factors, diagnosis, and treatment of peri-implantitis: A cross-cultural comparison of U.S. and European periodontists’ considerations. J. Periodontol..

[13] Saleh M.H.A., Dias D.R., Kumar P. (2024). The economic and societal impact of periodontal and peri-implant diseases. Periodontol. 2000.

[14] Schwarz F., Alcoforado G., Guerrero A., Jönsson D., Klinge B., Lang N., Mattheos N., Mertens B., Pitta J., Ramanauskaite A. (2021). Peri-implantitis: Summary and consensus statements of group 3. The 6th EAO Consensus Conference 2021. Clin. Oral. Implants. Res..

[15] Heitz-Mayfield L.J., Aaboe M., Araujo M., Carrión J.B., Cavalcanti R., Cionca N., Cochran D., Darby I., Funakoshi E., Gierthmuehlen P.C. (2018). Group 4 ITI Consensus Report: Risks and biologic complications associated with implant dentistry. Clin. Oral Implants Res..

[16] Garaicoa-Pazmino C., Couso-Queiruga E., Monje A., Avila-Ortiz G., Castilho R., Amo F. (2025). Disease Resolution Following the Treatment of Peri-implant Diseases: A Systematic Review. Int. J. Periodontics Restor. Dent..

[17] Fu J.H., Wang H.L. (2020). Breaking the wave of peri-implantitis. Periodontol. 2000.

[18] Alassy H., Parachuru P., Wolff L. (2019). Peri-implantitis diagnosis and prognosis using biomarkers in peri-implant crevicular fluid: A narrative review. Diagnostics.

[19] Bornes R., Montero J., Correia A., Marques T., Rosa N. (2023). Peri-implant diseases diagnosis, prognosis and dental implant monitoring: A narrative review of novel strategies and clinical impact. BMC Oral Health.

[20] Kabbashi S., Roomaney I.A., Chetty M. (2024). Bridging the gap between omics research and dental practice. BDJ Open.

[21] Sorsa T., Gursoy U.K., Nwhator S., Hernandez M., Tervahartiala T., Leppilahti J., Gürsoy M., Könönen E., Emingil G., Pussinen P.J. (2016). Analysis of matrix metalloproteinases, especially MMP-8, in gingival creviclular fluid, mouthrinse and saliva for monitoring periodontal diseases. Periodontol. 2000.

[22] Duarte P.M., Serrão C.R., Miranda T.S., Zanatta L.C.S., Bastos M.F., Faveri M., Figueiredo L.C., Feres M. (2016). Could cytokine levels in the peri-implant crevicular fluid be used to distinguish between healthy implants and implants with peri-implantitis? A systematic review. J. Periodontal Res..

[23] Carinci F., Romanos G.E., Scapoli L. (2019). Molecular tools for preventing and improving diagnosis of peri-implant diseases. Periodontol. 2000.

[24] Clish C.B. (2015). Metabolomics: An emerging but powerful tool for precision medicine. Mol. Case Stud..

[25] Gardner A., Carpenter G., So P.W. (2020). Salivary Metabolomics: From Diagnostic Biomarker Discovery to Investigating Biological Function. Metabolites.

[26] Hyvärinen E., Savolainen M., Mikkonen J.J.W., Kullaa A.M. (2021). Salivary Metabolomics for Diagnosis and Monitoring Diseases: Challenges and Possibilities. Metabolites.

[27] Page M.J., McKenzie J.E., Bossuyt P.M., Boutron I., Hoffmann T.C., Mulrow C.D., Shamseer L., Tetzlaff J.M., Akl E.A., Brennan S.E. (2021). The PRISMA 2020 statement: An updated guideline for reporting systematic reviews. Syst. Rev..

[28] Sterne J.A., Hernán M.A., Reeves B.C., Savović J., Berkman N.D., Viswanathan M., Henry D., Altman D.G., Ansari M.T., Boutron I. (2016). ROBINS-I: A tool for assessing risk of bias in non-randomised studies of interventions. BMJ.

[29] McGuinness L.A., Higgins J.P.T. (2021). Risk-of-bias VISualization (robvis): An R package and Shiny web app for visualizing risk-of-bias assessments. Res. Synth. Methods.

[30] Hamilton J. (2020). Investigation of Diagnostic and Prognostic Testing for Peri-Implantitis Using Quantitative Metabolomics. Master’s Thesis.

[31] Alassy H. (2021). Peri-Implantitis Prognosis Using Metabolomic Biomarkers in Peri-Implant Crevicular Fluid: A Longitudinal Study. Ph.D. Thesis.

[32] Liu Y., Yang H., Wang P., Shi Y., Shi R., Zhang S., Zhao Y., Lan J., Ge S. (2024). Correlation between short-chain fatty acids and peri-implant disease: A cross-sectional study. J. Periodontol..

[33] Song L., Lu H., Jiang J., Xu A., Huang Y., Huang J.P., Ding P.H., He F. (2024). Metabolic profiling of peri-implant crevicular fluid in peri-implantitis. Clin. Oral Implants Res..

[34] Fornasaro S., Rapani A., Farina F., Ibishi M., Pisnoli G., Stacchi C., Sergo V., Bonifacio A., Di Lenarda R., Berton F. (2023). Spectroscopic insights into peri-implant mucositis and peri-implantitis: Unveiling peri-implant crevicular fluid profiles using surface enhanced Raman scattering. Analyst.

[35] Mikkonen J.J.W., Singh S.P., Herrala M., Lappalainen R., Myllymaa S., Kullaa A.M. (2016). Salivary metabolomics in the diagnosis of oral cancer and periodontal diseases. J. Periodontal Res..

[36] Kouznetsova V.L., Li J., Romm E., Tsigelny I.F. (2021). Finding distinctions between oral cancer and periodontitis using saliva metabolites and machine learning. Oral Dis..

[37] Papale F., Santonocito S., Polizzi A., Giudice A.L., Capodiferro S., Favia G., Isola G. (2022). The New Era of Salivaomics in Dentistry: Frontiers and Facts in the Early Diagnosis and Prevention of Oral Diseases and Cancer. Metabolites.

[38] Baima G., Corana M., Iaderosa G., Romano F., Citterio F., Meoni G., Tenori L., Aimetti M. (2021). Metabolomics of gingival crevicular fluid to identify biomarkers for periodontitis: A systematic review with meta-analysis. J. Periodontal Res..

[39] Alamri M.M., Williams B., Le Guennec A., Mainas G., Santamaria P., Moyes D.L., Nibali L. (2023). Metabolomics analysis in saliva from periodontally healthy, gingivitis and periodontitis patients. J. Periodontal Res..

[40] Romano F., Meoni G., Manavella V., Baima G., Tenori L., Cacciatore S., Aimetti M. (2018). Analysis of salivary phenotypes of generalized aggressive and chronic periodontitis through nuclear magnetic resonance-based metabolomics. J. Periodontol..

[41] Heimisdottir L.H., Lin B.M., Cho H., Orlenko A., Ribeiro A.A., Simon-Soro A., Roach J., Shungin D., Ginnis J., Simancas-Pallares M. (2021). Metabolomics Insights in Early Childhood Caries. J. Dent. Res..

[42] Li K., Wang J., Du N., Sun Y., Sun Q., Yin W., Li H., Meng L., Liu X. (2023). Salivary microbiome and metabolome analysis of severe early childhood caries. BMC Oral Health.

[43] Schulz A., Lang R., Behr J., Hertel S., Reich M., Kümmerer K., Hannig M., Hannig C., Hofmann T. (2020). Targeted metabolomics of pellicle and saliva in children with different caries activity. Sci. Rep..

[44] Turi K.N., Romick-Rosendale L., Ryckman K.K., Hartert T.V. (2018). A review of metabolomics approaches and their application in identifying causal pathways of childhood asthma. J. Allergy Clin. Immunol..

[45] Chen Y., Li E.M., Xu L.Y. (2022). Guide to Metabolomics Analysis: A Bioinformatics Workflow. Metabolites.

[46] Zhang Y., Chen R., Zhang D.D., Qi S., Liu Y. (2023). Metabolite interactions between host and microbiota during health and disease: Which feeds the other?. Biomed. Pharmacother..

[47] Roberts L.D., Souza A.L., Gerszten R.E., Clish C.B. (2012). Targeted metabolomics. Curr. Protoc. Mol. Biol..

[48] Lu Y., Lin L., Ye J. (2022). Human metabolite detection by surface-enhanced Raman spectroscopy. Mater. Today Bio..

[49] Premasiri W.R., Lee J.C., Sauer-Budge A., Théberge R., Costello C.E., Ziegler L.D. (2016). The biochemical origins of the surface-enhanced Raman spectra of bacteria: A metabolomics profiling by SERS. Anal. Bioanal. Chem..

[50] Robitaille N., Reed D.N., Walters J.D., Kumar P.S. (2016). Periodontal and peri-implant diseases: Identical or fraternal infections?. Mol. Oral Microbiol..

[51] Sahrmann P., Gilli F., Wiedemeier D.B., Attin T., Schmidlin P.R., Karygianni L. (2020). The Microbiome of Peri-Implantitis: A Systematic Review and Meta-Analysis. Microorganisms.

[52] Ferreira S.D., Martins C.C., Amaral S.A., Vieira T.R., Albuquerque B.N., Cota L.O.M., Lima R.P.E., Costa F.O. (2018). Periodontitis as a risk factor for peri-implantitis: Systematic review and meta-analysis of observational studies. J. Dent..

[53] Darby I. (2022). Risk factors for periodontitis & peri-implantitis. Periodontol. 2000.

[54] Dalago H.R., Schuldt Filho G., Rodrigues M.A.P., Renvert S., Bianchini M.A. (2017). Risk indicators for Peri-implantitis. A cross-sectional study with 916 implants. Clin. Oral Implants Res..

[55] Guan X., Li W., Meng H. (2021). A double-edged sword: Role of butyrate in the oral cavity and the gut. Mol. Oral Microbiol..

[56] Brito F., Curcio H.F.Q., da Silva Fidalgo T.K. (2022). Periodontal disease metabolomics signatures from different biofluids: A systematic review. Metabolomics.

[57] Na H.S., Kim S., Yu Y., Kim S.Y., Kim H.J., Lee J.Y., Lee J.H., Chung J. (2021). Molecular subgroup of periodontitis revealed by integrated analysis of the microbiome and metabolome in a cross-sectional observational study. J. Oral Microbiol..

[58] Kim S., Kim H.J., Song Y., Lee H.A., Kim S., Chung J. (2021). Metabolic phenotyping of saliva to identify possible biomarkers of periodontitis using proton nuclear magnetic resonance. J. Clin. Periodontol..

[59] Leonov G.E., Varaeva Y.R., Livantsova E.N., Starodubova A.V. (2023). The Complicated Relationship of Short-Chain Fatty Acids and Oral Microbiome: A Narrative Review. Biomedicines.

[60] Basic A., Dahlén G. (2023). Microbial metabolites in the pathogenesis of periodontal diseases: A narrative review. Front. Oral Health.

[61] Kuboniwa M., Sakanaka A., Hashino E., Bamba T., Fukusaki E., Amano A. (2016). Prediction of Periodontal Inflammation via Metabolic Profiling of Saliva. J. Dent. Res..

[62] Sakanaka A., Kuboniwa M., Katakami N., Furuno M., Nishizawa H., Omori K., Taya N., Ishikawa A., Mayumi S., Isomura E.T. (2021). Saliva and Plasma Reflect Metabolism Altered by Diabetes and Periodontitis. Front. Mol. Biosci..

[63] Sakanaka A., Kuboniwa M., Hashino E., Bamba T., Fukusaki E., Amano A. (2017). Distinct signatures of dental plaque metabolic byproducts dictated by periodontal inflammatory status. Sci. Rep..

[64] Rashid M.H., Yellarthi S.P.K., Yellarthi P.K., Didugu B.G.L., Mamillapalli A. (2024). Combined assessment of lysine and N-acetyl cadaverine levels assist as a potential biomarker of the smoker periodontitis. Amino Acids.

[65] Andörfer L., Holtfreter B., Weiss S., Matthes R., Pitchika V., Schmidt C.O., Samietz S., Kastenmüller G., Nauck M., Völker U. (2021). Salivary metabolites associated with a 5-year tooth loss identified in apopulation-based setting. BMC Med..

[66] Barnes V.M., Ciancio S.G., Shibly O., Xu T., Devizio W., Trivedi H.M., Guo L., Jonsson T.J. (2011). Metabolomics Reveals Elevated Macromolecular Degradation in Periodontal Disease. J. Dent. Res..

[67] Qi H., Li B., Wang H., Cai Q., Quan X., Cui Y., Meng W. (2018). Effects of d-valine on periodontal or peri-implant pathogens: Porphyromonas gingivalis biofilm. J. Periodontol..

[68] Liebsch C., Pitchika V., Pink C., Samietz S., Kastenmüller G., Artati A., Suhre K., Adamski J., Nauck M., Völzke H. (2019). The Saliva Metabolome in Association to Oral Health Status. J. Dent. Res..

[69] Citterio F., Romano F., Meoni G., Iaderosa G., Grossi S., Sobrero A., Dego F., Corana M., Berta G.N., Tenori L. (2020). Changes in the salivary metabolic profile of generalized periodontitis patients after non-surgical periodontal therapy: A metabolomic analysis using nuclear magnetic resonance spectroscopy. J. Clin. Med..

[70] Barnes V.M., Teles R., Trivedi H.M., Devizio W., Xu T., Mitchell M.W., Milburn M.V., Guo L. (2009). Acceleration of purine degradation by periodontal diseases. J. Dent. Res..

[71] Phang J.M., Pandhare J., Liu Y. (2008). The Metabolism of Proline as Microenvironmental Stress Substrate. J. Nutr..

[72] Karna E., Szoka L., Huynh T.Y.L., Palka J.A. (2019). Proline-dependent regulation of collagen metabolism. Cell Mol. Life Sci..

[73] Fouillen A., Mary C., Ponce K.J., Moffatt P., Nanci A. (2021). A proline rich protein from the gingival seal around teeth exhibits antimicrobial properties against Porphyromonas gingivalis. Sci. Rep..

[74] Jayasinghe T.N., Harrass S., Erdrich S., King S., Eberhard J. (2022). Protein Intake and Oral Health in Older Adults-A Narrative Review. Nutrients.

[75] García-Villaescusa A., Morales-Tatay J.M., Monleón-Salvadó D., González-Darder J.M., Bellot-Arcis C., Montiel-Company J.M., Almerich-Silla J.M. (2018). Using NMR in saliva to identify possible biomarkers of glioblastoma andchronic periodontitis. PLoS ONE.

[76] Wang Y., Deng P., Liu Y., Wu Y., Chen Y., Guo Y., Zhang S., Zheng X., Zhou L., Liu W. (2020). Alpha-ketoglutarate ameliorates age-related osteoporosis via regulating histone methylations. Nat. Commun..

[77] Liu R., Gao Y., Huang L., Shi B., Yin X., Zou S. (2023). Alpha-ketoglutarate up-regulates autophagic activity in peri-implant environment and enhances dental implant osseointegration in osteoporotic mice. J. Clin. Periodontol..

[78] Chen L., Li Q., Ma S., Wang B. (2024). α-Ketoglutarate promotes autophagic activity under a peri-implant condition to enhance osseointegration of dental implant in rats with osteoporosis. Connect. Tissue Res..

[79] Li Y., Liu L., Li Y., Song W., Shao B., Li H., Lin W., Li Q., Shuai X., Bai M. (2023). Alpha-ketoglutarate promotes alveolar bone regeneration by modulating M2 macrophage polarization. Bone Rep..

